# Heart in the ‘jaws’ of a constrictor, unusual cause of subacute right heart failure

**DOI:** 10.1186/s13019-019-0916-9

**Published:** 2019-06-15

**Authors:** Geoffrey Joseph Changwe, Zhang Wenlong, Haizhou Zhang, Chengwei Zou

**Affiliations:** Departments of Cardiovascular Surgery and Imaging, Shandong Provincial Hospital, Affiliated to Shandong University, No.324 –Jingwu, Jinan, People’s Republic of China

**Keywords:** Constrictive pericarditis, Right heart failure, Echocardiography

## Abstract

**Background:**

Constrictive Pericarditis(CP) can be viewed as a constellation of syndromes resulting from compression of the heart, etiologies, course and types are well discussed in other reports. However, localized CP as a cause of right heart failure is rare, and presentation with interposed fluid under-pressure is extremely odd.

**Case report:**

A case of C.X. Z, male aged 39 years old, who presented to our department with sudden onset of symptoms of subacute right heart failure due localized CP. In January, 2018 C.X.Z presented to the county hospital with complaints of 10-day history of transient mild bilateral pedal edema. He was managed on diuretic therapy and symptoms resolved completely. 10 months later, he suddenly presented to the local facility with symptoms of subacute right heart failure. 7 days after on-set of symptoms, his condition shifted from NYHA I to III-IV. Although wake-up chest radiography appeared normal, standard medical therapy yielded no positives results. He was referred to our hospital, upon which after echocardiography and computed tomography investigations, aforementioned diagnosis was made. We performed off-pump partial pericardiectomy with no complications. After operation, he received analgesics and diuretics for pain and edema(ascites) respectively. He was discharged 7-days after operation on analgesics only, with no symptoms of right heart failure.

**Conclusion:**

Localized constrictive pericarditis as a cause of subacute right heart failure (RHF) has never been reported elsewhere, and presentation with interposed fluid is extremely odd. Progressive symptoms of Acute RHF in the absence of typical radiologic clue ‘egg-shell’ should heighten suspicion index of CP.

**Electronic supplementary material:**

The online version of this article (10.1186/s13019-019-0916-9) contains supplementary material, which is available to authorized users.

## Background

Constrictive Pericarditis (CP) is a potentially curable cause of diastolic heart failure. Known and common etiologies include previous heart surgery, chest radiation, trauma and infections such as tuberculosis. Conversely, a proportion of the cases may not have well identifiable causes, thus deemed idiopathic. Early identification is cardinal, as timely pericardiectomy is associated with lower operative complications. Clinically, timely diagnosis can be challenging especially in the absence of radiological signs. With high suspicion index, careful evaluation of jugular venous pressure and its wave forms aids diagnosis. Additionally, modern echocardiography machines in experienced hands permit confirmation of clinically suspected in majority cases.

## Case presentation

A case of C.X.Z, male aged 39-year old, and farmer by profession was wheeled into our department with severe symptoms of subacute RHF. His spouse narrated that in January,2018, he had experienced mild bilateral swelling of lower limbs (in form of stockings), and was managed on diuretics for about 7 days, after which symptoms disappeared completely. She denied him having had any cardiac surgery, chest radiation, tuberculosis or significant chest trauma. 8 months after initial symptoms, thus in October,2018, he suddenly developed chest pain, which he thought was due to long working hours in the field. On-counter remedies (pain killers) offered temporal relief. After 2-days of progressive chest pain, patient begun experiencing abdominal discomfort and observed swelling of feet after bed. On the 4th day in his illness, he developed shortness, a development that prompted him seek medical attention.

On presentation the patient through his spouse complained of breathing difficulties, abdominal fullness and swelling of lower limbs. She further narrated that, during bed time, shortness of breath worsened upon lying flat. During physical examination, patient exhibited incoherent talk, responded to various questions with same answer repeatedly. Both the neck veins (JVD~ > 15mmH2O) and abdomen were highly distended. Chest auscultation demonstrated a ‘cardiac knock’, and both S_1_ and S_2_ were muffled. Abdominal palpation revealed gross ascites. The lower extremities were cold to touch with bilateral pitting edema from knee and below. Prior and post procedure vitals are tabulated in Table.1.

**Table 1:** Pre−/post operation hemodynamic and respiratory indices

Variable	Respiration	Pulse	Blood pressure	Ejection fraction
Pre-operation	32 r/m	160b/m	78/40 mmHg	50%
Post operation	20r/m	84b/m	130/62 mmHg	61%
Respirations per minute,r/m.beats per minute/millimeter Mercury,mmHg.				

### Diagnostic Procedures

**Fig. 1:**
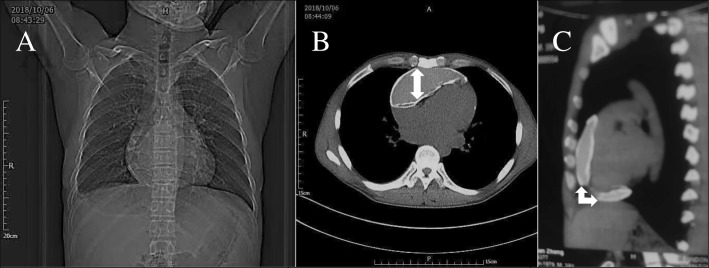
Imaging results, Panel A chest radiography, panels: B & C CTA. Panel A. Chest radiography, Panels B and C CTA with solid arrows demonstrating calcification & fluid


Diagnosis of localized CP was established using cogent imaging results of comprehensive transthoracic echocardiography (TTE) and computed tomography(CTA). A 4 chamber video clip (Additional file 1) of a 2D TTE examination demonstrates dyskinesia of the right ventricle(RV) due to the presence of a thickened (calcified) pericardium(cyst-like) on its anterior wall. Other visible abnormalities include: right atrium enlargement, respirophasic interventricular deviation into left ventricle and shudder, scientifically proven to be due to the differential filling rates of both ventricles in diastole. A comprehensive TTE report (not shown here) demonstrated hepatic vein distension, a patent inferior vena cava (with no respirophasic variation) and ejection fraction(EF) of 50%. The chest radiography appeared normal (Panel A). However, CTA uncovered two adjacent pads of calcification with interposed fluid: panels B and C (Fig. 1)**.**Because CP is relatively common in our region,radiology personnel have gained experience in diagnosing CP with occasional application of the Swan-Ganz catheter.

Additional file 12D 4CTTE video. (AVI 11827 kb)


### Decortication

**Fig. 2:**
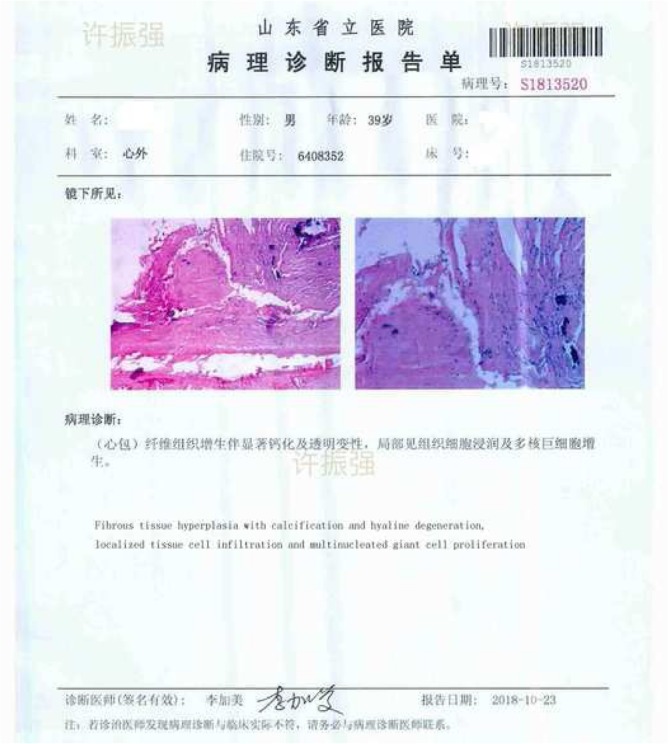
Pericardium tissue pathological report

Through standard midline sternotomy, an off-pump partial pericardiectomy was performed from cardiac surgery operation room. Using a combination of cautery, scissors and sharp hooks, we approached the pericardium from the free RV wall (junction with right atrium). After 30 to 40 min into decortication process, we judiciously punctured a plate of calcified tissue overlaying the anterior RV wall with subsequent gush of a ῾milk-like’ fluid(approx.700mls). Fluid decompression led to adjustments of ventriculae pressures and cardiac out-put. The video in (Additional file 2) shows the heart beating with less obstruction. Fluid sample results and pericardium tissue pathological report are depicted in (Table 2) and (Fig. 2) respectively.

**Table 2:**
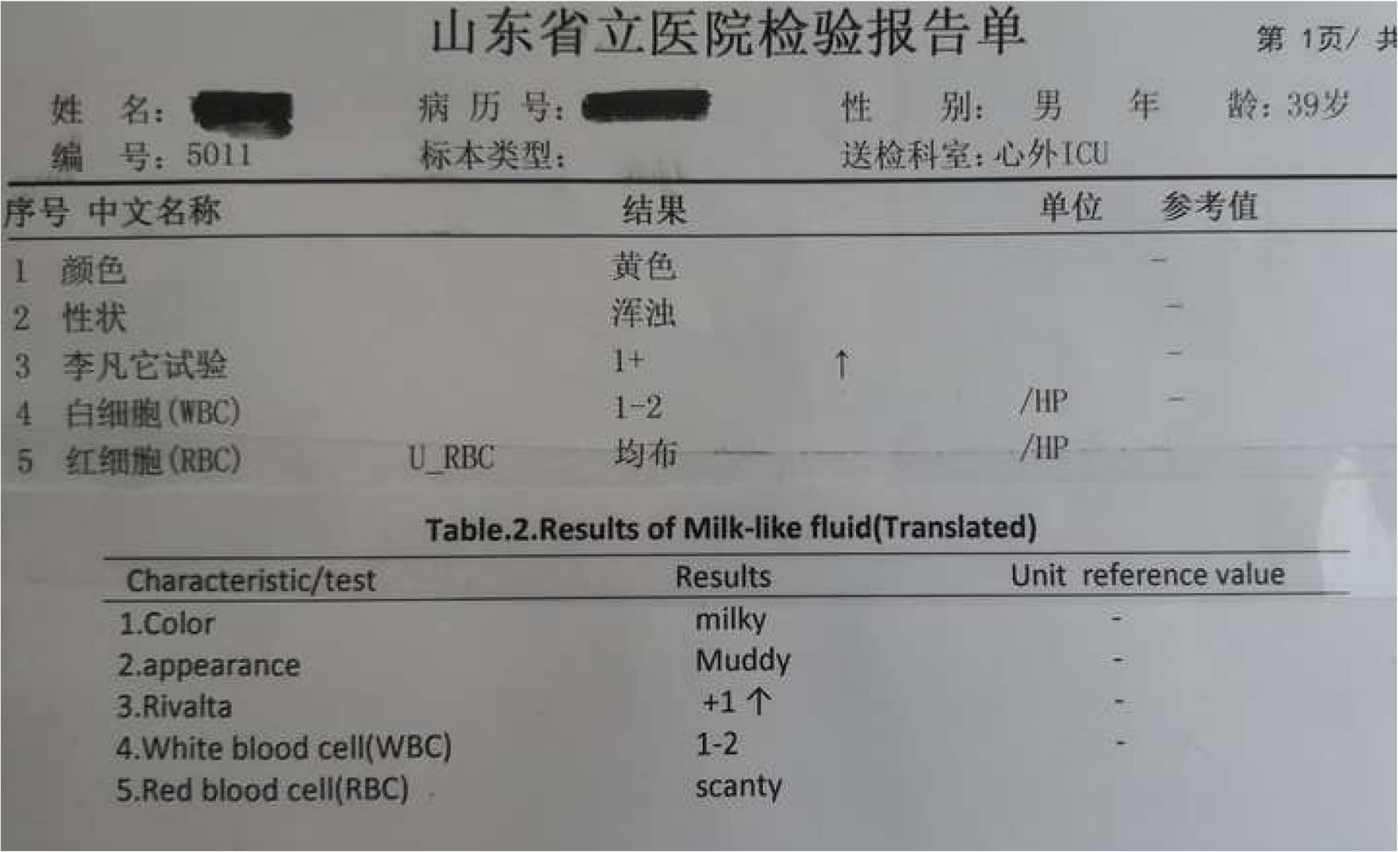
milk-like fluid analysis report

## Discussion

Constrictive pericarditis(CP) can be viewed as a constellation of syndromes resulting from compression of the heart, etiology course and types are well discussed in other reports [1, 2]. However, localized CP, as a cause of acute right heart failure(RHF) is rare, and presentation with interposed fluid under-pressure is extremely odd [3, 4]. Our case well presented in both still images and video, demonstrate two adjacent pads responsible for pathological symptoms. When viewed from apical angle in sagittal plane, the two pads assume the shape of two jaws mouth swallow the heart, hence, the title “Heart in the ‘JAWS’ of a constrictor’ ‘We acknowledge that this particular case is worthy differentiating from pectus excavatum [5].

Our case was unusual in both course and imaging presentation. Usually, CP is known to take a chronic course, and medical therapy take a palliative role in majority cases. Surprisingly enough, the right heart pump was suddenly failing in someone with a very unremarkable past medical history. The chest radiography did not show obvious radiological clue ‘egg-shell’ commonly observed in majority cases. At this juncture, we can only appreciate the advancement in imaging modalities that timely diagnosis was made.

Pericardiectomy(partial/complete) is associated with a myriad complications including death. In a recent European study (Busch et al.) RV dilation and EF < 55% were cited as risk factors for(early/late) mortality after pericardiectomy for chronic CP. Despite EF of 50% the patient experienced spontaneous recovery of cardiac functions due to normal ventriculae dimensions and absence of associated lesions (tricuspid /septal). Post operation therapy was centered on pain management and liquidation of edema and ascites using low doses of Spironolactone and Furosemides. The patient was discharged home free of symptoms (NYHA-Class I) 7 days after operation.

## Conclusion

In view of both course and presentation, when faced with acute RHF in the absence of chest radiographic signs of CP, it’s suspicion of index must be heightened and advanced imaging modalities sought. Otherwise, patient could be subjected to medical therapy with catastrophic outcome. We believe this case is very unusual and could aid accurate diagnosis of similar cases.

## Additional files

Additional file 2: Video-Immediate post fluid decompression. (FLV 1567 kb)

## Data Availability

Available on appropriate request from author.

## Abbreviations

CP: Constrictive pericarditis
CT: Computed tomography angiography
EF: Ejection fraction
RHF: Right heart failure
TTE: Transthoracic echocardiography
